# Synovial Chondromatosis in a Rural Healthcare Setting

**DOI:** 10.7759/cureus.34498

**Published:** 2023-02-01

**Authors:** Shafneed Chaliyadan, Apoorva Gujar, Sharon Vallikkad, Raman Kataria

**Affiliations:** 1 General Surgery, Jan Swasthya Sahyog, Bilaspur, IND

**Keywords:** clinical case report, knee joints, joint restriction, healthcare in rural india, resource limited setting, joint swelling, synovial osteochondromatosis

## Abstract

Synovial chondromatosis is a rare, benign, and metaplastic cause of joint swelling resulting in the formation of cartilaginous nodules in the joint space. It is usually an oligoarticular disorder of large joints that typically manifests in the third to fifth decade of life. Synovial chondromatosis can be primary or secondary depending on whether an underlying etiology can be identified. Diagnosis can be made using imaging studies of the affected joint and confirmed on histopathology. Management of synovial chondromatosis can be done arthroscopically or surgically.

We present a case of a 23-year-old male who presented with a long history of right knee pain, swelling, and limitation in range of motion. An X-ray of the knee showed multiple intra-articular and soft tissue calcifications. Due to the limitations of our setting, we proceeded with an open biopsy. During arthrotomy, clear straw-colored fluid with multiple nodules of varied sizes was found. A google image search helped put us in the direction of the diagnosis of synovial chondromatosis. We did a complete evacuation of loose bodies and a biopsy of synovium, which confirmed the diagnosis.

The rarity of synovial chondromatosis results in a delay in the diagnosis. With the thoughtful application of resources and surgical principles, synovial chondromatosis can be safely and effectively managed in resource-limited settings.

## Introduction

Synovial chondromatosis is a rare cause of joint swelling caused by benign metaplasia of synovial connective tissue of joints, bursae, or tendon sheaths, into cartilage and detachment of these nodules, or loose bodies, into the joint space. It typically affects the large single joints like the knee, ankle, hip, elbow, and shoulder but can rarely involve smaller joints as well [1-4]. Synovial chondromatosis, like other arthropathies, presents with joint pain, swelling, and stiffness but is characterized by crepitus on joint movement.

The disease usually presents in the third to fifth decade of life but is also rarely reported in children and adolescents [5-7]. Synovial chondromatosis can be classified into primary and secondary. Primary synovial chondromatosis is of unknown etiology, whereas secondary synovial chondromatosis may occur following trauma or in arthritis. Synovial chondromatosis is diagnosed using a plain radiograph, CT scan, or MRI, and definitive confirmation is made on histopathological examination. The entity is treated arthroscopically or surgically and is aimed to alleviate symptoms and prevent joint destruction and disability.

We present a case of synovial chondromatosis from a rural healthcare setting in central India and our approach to managing it.

## Case presentation

**Figure 1:**
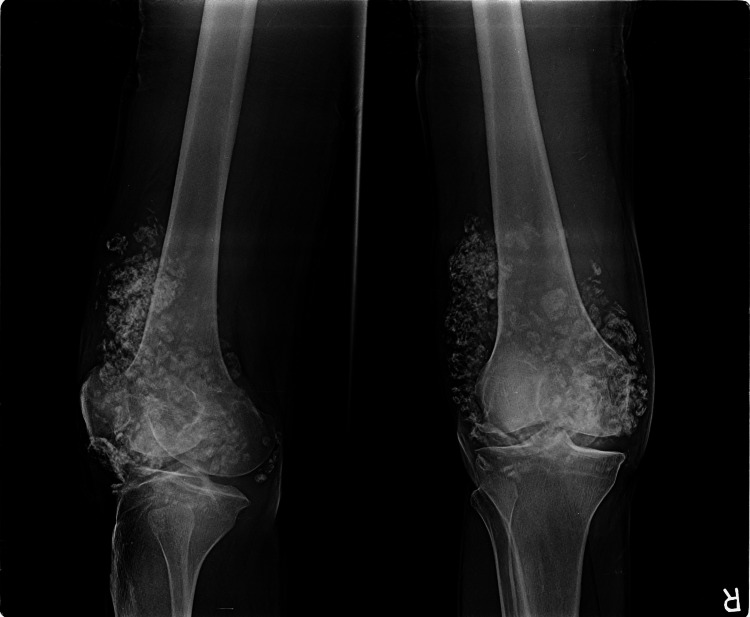
X-ray of the right knee, lateral and AP views. Note the presence of multiple intraarticular calcifications.

A 23-year-old male, a daily wage laborer, presented to the outpatient department of our rural hospital in Chhattisgarh, India, with right knee pain for the past three years, with associated swelling and limitation in range of motion. The pain was exacerbated with flexion of the joint. He gave a history of falls from a two-wheeler three years ago with no obvious injuries. Aspiration of the right knee joint was done elsewhere one year ago and reportedly had blood-mixed content. There was no history of fever or discharge from the joint. On examination, he had a 10x8cm diffuse swelling involving the right knee joint, non-erythematous, soft, non-tender, with crepitus and palpable multiple hard nodules of varying size. His knee flexion was limited to 30°. He underwent an X-ray of the right knee, which showed multiple intra-articular and soft tissue calcifications of variable size and shape with relatively preserved joint spaces. Visualized bones showed degenerative changes in the form of osteophytic growth, tibial spiking, and endplate sclerosis. No bony erosion was seen (Figure 1). No further imaging was carried out due to logistical and financial constraints. Differential diagnoses of synovial chondrosarcoma, synovial hemangioma, and tumoral calcinosis were kept in mind, and an open biopsy was planned.

**Figure 2:**
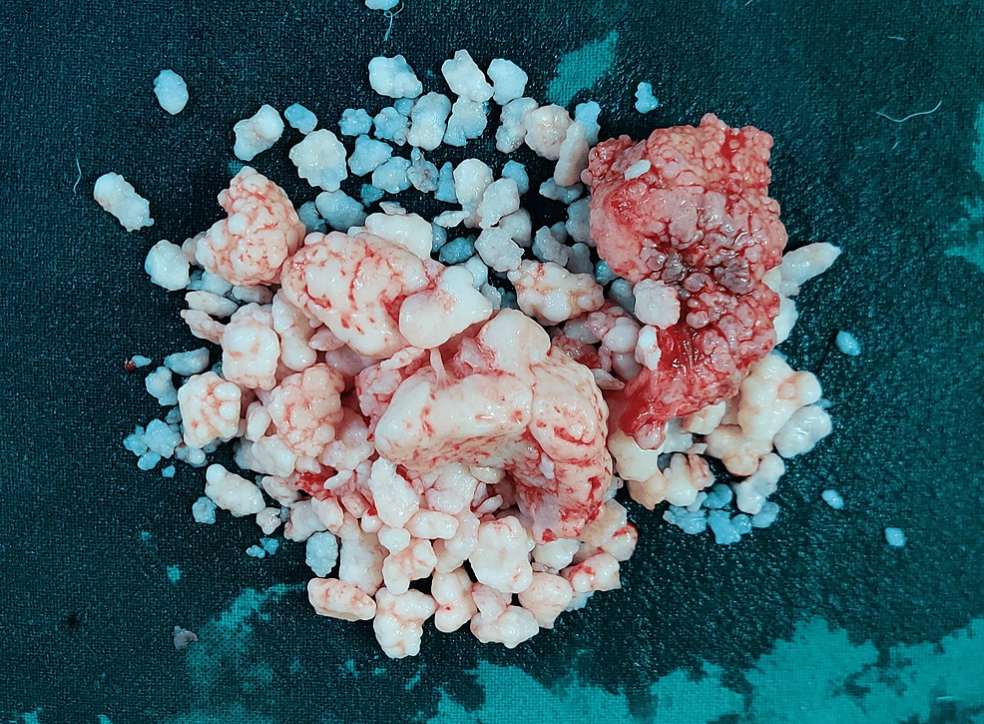
Rice bodies/joint mice, intra-articular nodules removed from the right knee synovial cavity.

During arthrotomy, clear straw-colored fluid with multiple hard rice bodies was seen (Video 1). Nodules varied in size from 0.5 cm to 5.0 cm (Figure 2). Articular surfaces of the joint were smooth, but the synovium was thickened. Some nodules were found to be firmly adherent and arising from the synovial lining. Curious about our findings, we did an image search of the specimen on Google Images, and it directed us toward the diagnosis of synovial chondromatosis. A literature review on synovial chondromatosis was correlating with our patient’s clinical history, examination, X-ray, and intra-operative findings. A complete evacuation of all free loose bodies was carried out with a biopsy of the synovium. Arthrotomy was closed with a drain. The patient was discharged after an uneventful postoperative course. Histopathology confirmed the diagnosis of synovial chondromatosis. On histopathology, multiple portions of synovial tissues showed cartilaginous neoplasms composed of lobules of myxo hyaline cartilage with foci of enchondral ossification and bland-looking chondrocytes. There was no evidence of increased cellularity, mitotic activity, or necrosis. He has since resumed his work and has been symptom-free for the past six months.

**Video 1:** Intra-operative video showing right knee arthrotomy.

## Discussion

Synovial chondromatosis is a rare, benign, metaplastic, usually oligoarticular disorder, most commonly occurring in knee joints. There are intra-articular nodules formed from the synovium, which become free and can get secondarily ossified [8-11]. The disease has a male predisposition [8]. The disease is usually progressive, leading to joint destruction, but spontaneous regression has also been reported [12]. Progression of synovial chondromatosis follows three recognizable phases; initially, there is active synovitis without loose bodies, followed by a transitional phase of recognizable intraarticular loose bodies, which are cartilaginous and active intra-synovial proliferation. Lastly, there are multiple ossified loose bodies without active synovitis [13]. In the early phases of the disease, patients can be managed conservatively with NSAIDs and intra-articular steroid injections. Surgical management is warranted when there is persistent swelling and restricted motion. Removal of loose bodies alone provides symptomatic relief, but there is potential for recurrence [14]. Removal of loose bodies with anterior and posterior synovectomy is the treatment of choice with the least recurrence [15]. The same can be achieved arthroscopically. Arthroscopic approaches are successful and result in less post-operative joint stiffness. Recurrence after arthroscopy can also be managed arthroscopically [16,17]. Radiosynovectomy with 188 re-tin colloid was explored as adjuvant therapy in synovial chondromatosis with success, facilitating surgical removal and potentially reducing the risk of recurrence [18]. The disease can rarely undergo malignant transformation into chondrosarcoma [19,20].

## Conclusions

Synovial chondromatosis is a rare cause of knee swelling and maybe only rarely encountered in one’s practice. The disease can be safely managed in a rural setting with limited resources, with a thoughtful application of available technologies and basic surgical principles.
